# Systemic Inflammatory Changes in Spinal Cord Injured Patients after Adding Aquatic Therapy to Standard Physiotherapy Treatment

**DOI:** 10.3390/ijms25147961

**Published:** 2024-07-21

**Authors:** María. Teresa Agulló-Ortuño, Helena Romay-Barrero, Johan Lambeck, Juan M. Blanco-Calonge, Rubén Arroyo-Fernández, Paula Richley Geigle, Raquel Menchero, Gonzalo Melgar del Corral, Inés Martínez-Galán

**Affiliations:** 1 Department of Nursing Physiotherapy and Occupational Therapy, Faculty of Physical Therapy and Nursing, University of Castilla-La Mancha, Avda. Carlos III s/n, 45071 Toledo, Spain; mariateresa.agullo@uclm.es (M.T.A.-O.); juanmanuel.blanco@uclm.es (J.M.B.-C.); ruben.arroyo@uclm.es (R.A.-F.); gonzalo.melgar@uclm.es (G.M.d.C.); ines.martinez@uclm.es (I.M.-G.); 2 Research Group on Water and Health (GIAS), University of Castilla-La Mancha. Avda. Carlos III s/n, 45071 Toledo, Spain; raquel.menchero@uclm.es; 3 Association International Aquatic Therapy Faculty, 7324 Valens, Switzerland; lambeck.hydro@freeler.nl; 4 The Western North Carolina VA Health Care System (WNCVAHCS), Asheville, NC 28805, USA; geigle@comcast.net

**Keywords:** spinal cord injury, systemic inflammation, cytokines, physiotherapy, aquatic physiotherapy

## Abstract

Spinal cord injury (SCI) is a severe medical condition resulting in substantial physiological and functional consequences for the individual. People with SCI are characterised by a chronic, low-grade systemic inflammatory state, which contributes to further undesirable secondary injuries. This study aimed to evaluate the effect of adding aquatic therapy to the standard physiotherapy treatment, implemented in two different schedules, on systemic inflammation in SCI patients. Additionally, the relationship between cytokine blood levels and changes in functionality (measured with the 6MWT, 10MWT, WISCI, BBS, and TUG tests) throughout the study was assessed. A quantitative multiplexed antibody assay was performed to measure the expression level of 20 pro- and anti-inflammatory cytokines in blood samples from SCI patients at three time points: baseline, week 6, and immediately post-intervention (week 12). This study identified a complex signature of five cytokines (IL-12p70, IL-8, MCP-1, IL-1α, and IP10) associated with the time course of the two physiotherapy programs. Two other cytokines (IL-4 and TNF-α) were also associated with the functional recovery of patients. These could be important indicators for SCI prognosis and provide a basis for developing novel targeted therapies.

## 1. Introduction

Spinal cord injury (SCI) can be an overwhelming condition, traditionally considered incurable, with a major impact on the individual’s life. Additionally, it is the second most common cause of paralysis, after stroke [1,2]. Regardless of the origin and classification, SCI symptoms vary widely depending on the injury location and severity of damage, as well as the adherence to treatment. Paralysis of the lower limbs, whether incomplete or complete, may hinder or limit walking. Furthermore, related complications may include muscle atrophy, loss of voluntary motor control, spasticity, infections, pressure ulcers, and chronic pain, among others. According to the World Health Organization (WHO), global estimates suggest that in 2021, approximately 15.4 million people were living with SCI, although incidence and prevalence vary across countries. Moreover, data reported from low-income countries, where medical and rehabilitation services are often limited, are frequently under reported [3]. Trend projections up to 2030 reveal a slight decrease in age standardized incidence (ASIR) for males, an upward trend in age-specific incidence rates for both sexes, and a similar pattern in age-standardized YLD rates [4]. Despite the relatively low incidence of SCI, this condition poses a very high cost and substantial financial burden on healthcare systems, mainly due to the complex medical assistance and chronic care required, in addition to economic consequences derived from productivity losses [5]. The mortality risk, which depends on the availability of quality and timely clinical care and rehabilitation services, also differs widely between countries. Historically, SCI has been related to very elevated mortality rates, mainly due to inappropriate management of SCI-related impairment and secondary conditions [3]. However, the current increase in cases of incomplete SCI and the extended life expectancy of these individuals shifted the focus from prevention and cure to the restoration of mobility and optimisation of functional capacities. Nowadays, the gold standard for documenting the level and severity of SCI is the International Standards for Neurological Classification of Spinal Cord Injury (ISNCSCI), published by the American Spinal Injury Association (ASIA) [6].

Therapeutic advances in spinal cord care attained over the last decade are highly promising. In this line, aquatic therapy (AP) is often incorporated in the treatment or rehabilitation protocols of SCI patients with the overall goal of improving mobility. AP incorporates the mechanical and thermal characteristics of water during partial or complete immersion. In patients with a deranged biological system, AP evokes short-term and long-term adaptation mechanisms by means of specific stimuli that induce biological changes with therapeutic effects [7,8]. Thereby, aquatic interventions in SCI patients should be multimodal and interdisciplinary, as the immersion in water elicits a wide range of physiologic, emotional, and cognitive reactions such as pain relief, blood flow, edema, connective tissue extensibility, changes in tissue metabolism, or inflammation, resulting mainly from the effects of hydrostatic pressure and water temperature [9,10,11]. Consequently, AP combines the benefits of hydrotherapy plus those of standard rehabilitation.

AP is frequently included in the treatment or rehabilitation protocols for individuals experiencing SCI. Nevertheless, the available research on the systemic biochemical effects of these treatments is limited. SCI causes immediate, recognisable damage followed by a secondary state of tissue degeneration that can develop over a variable time span. The mechanisms underlying this secondary injury are multiple and not fully understood at present. Evidence suggests systemic low-grade inflammation contributes to this damage and the balance between pro-inflammatory and anti-inflammatory agents play a critical role in the progression and outcome of the lesion [12,13]. In the specific case of SCI, the pro-inflammatory profile of these individuals is favoured by diverse risk factors, such as reduced lean muscle mass, physical inactivity, and adipose accumulation. The inflammatory response to exercise involves acute-phase protein and cytokine responses, making the relationship between these molecules and the effects of physiotherapy treatments particularly relevant, especially in SCI patients who exhibit a notable inflammatory component [14,15,16,17].

Cytokines and chemokines belong to a large family of small signaling proteins with pleiotropic effects in human physiology. They are synthesised by a variety of cells, including microglia, endothelial, and immune cells. While cytokines demonstrate physiological and neuromodulatory functions in the central nervous system, they can also contribute to neuronal damage when concentrations are excessive, unbalanced, or persistent. The elevated synthesis and release of pro-inflammatory mediators immediately following the occurrence of a SCI has been implicated in secondary degeneration [18]. Additionally, several cytokines, growth factors, and neurotrophins are involved in angiogenesis, neuron survival, and the regeneration process after the injury [13,19]. Although these molecules primarily function near the tissues where they are secreted, their systemic release also influences cell survival, chemotaxis, proliferation, and differentiation. Consequently, individuals with SCI often exhibit enhanced systemic low-grade inflammation, an uncontrolled chronic situation that potentially contributes to increased mortality and comorbidity, as well as higher risks for cardiovascular and respiratory disease, which are common in these patients [20].

**Figure 1:**
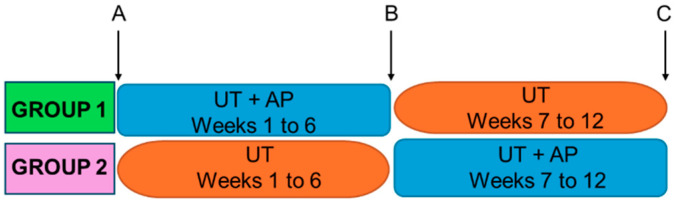
Graphical representation of the experimental design of the study. Participants were randomised into two groups. Group 1 first performed aquatic physiotherapy (AP) combined with the usual physiotherapy treatment (UT) for 6 weeks, followed by 6 weeks of UT alone; Group 2 initially underwent 6 weeks of UT alone followed by 6 weeks of AP combined with UT. Collection of blood samples and functional analyses of patients were performed at time points A, B, and C in both groups.

The effect of a physiotherapy treatment may also be conditioned by the moment in which it is applied. Thus, we propose the study of two different intervention programs: one in which patients first perform AP together with the usual physiotherapy treatment (UT), and then continue only with UT; and another in which patients first perform UT and then UT combined with AP (Figure 1).

The primary purpose of this study was to assess the effectiveness of incorporating AP in the UT, performed in two different intervention schedules, on the systemic inflammatory state of SCI patients. As a secondary exploratory aim, the relationship between cytokine levels and changes in functionality throughout the study was also evaluated.

## 2. Results

### 2.1. Experimental Design and Patients

Figure 1 shows the study experimental design. Patients were randomised into two treatment groups: those in Group 1 underwent 6 weeks of AP combined with their UT followed by 6 weeks of UT alone; the patients in Group 2 received the UT for the first 6 weeks followed by 6 weeks of a combination of UT and AP. A total of 40 patients completed the study, 21 from Group 1 and 19 from Group 2.

The anthropometric and socioeconomic characteristics of the participants are summarised in Table 1. No statistically significant differences were found between both treatment groups for sex, age, employment status, and educational level. In contrast, inter-group differences were observed for marital status. However, this was likely due to chance and does not hold special significance regarding the obtained results. Additionally, no statistically significant differences were noted between groups in terms of body mass index (BMI), injury type, or the ASIA Impairment Scale (AIS) classification (Table 1).

### 2.2. Inflammatory Mediators at Different Time Points of the Study (A, B, and C)

Cytokine plasma levels were measured at three time points of the physiotherapy programs: upon inclusion in the study (point A), at week 6, namely after Group 1 finished the combined treatment (UT + AP) and Group 2 finished the UT (point B), and immediately post-intervention at week 12 (point C) (Figure 1).

No statistically significant differences in cytokine levels were found when we compared groups 1 and 2 at any of the evaluation points (A, B, or C). However, we must point out that there was great variability in these values in each of the treatment groups.

**Figure 2:**
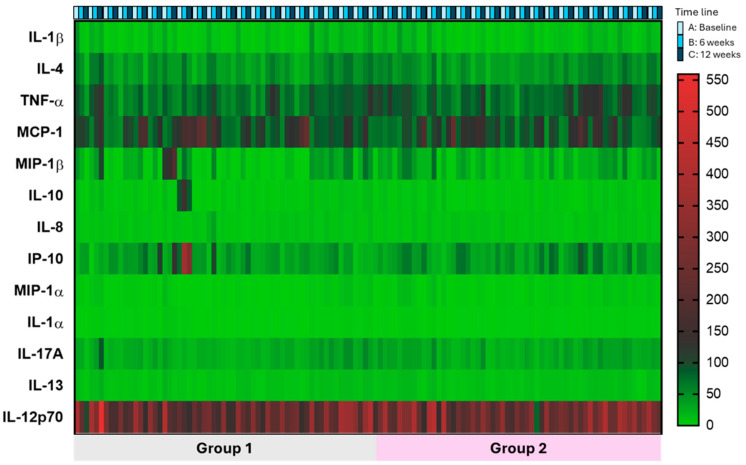
Heatmap representing the variations in cytokine levels for each of the patients at the three assessment points.

In Group 1, statistically significant differences were found at time point C compared to point A in the following cytokine levels: CD26P, CD62E, MCP-1, IL-12p70, IL-10, IL-8, IP-10, IL-1α, and IL-13. Similarly, significant differences were observed at time point B versus point A in the following cytokine levels: CD26P, CD62E, MCP-1, MIP-1β, TNF-α, GM-CSF, IL12-p70, IL-8, IP-10, IL-1β, IL-17A, and IL-13. Comparing cytokine levels at time point C versus point B, statistically significant differences were found in: CD26P, IL-1 β, IL-4, and TNF-α (Supplementary Table S1). Figure 2 shows a representative heatmap of these data.

In Group 2, statistically significant differences were found at time point C compared to point A in the following cytokine levels: CD26P, CD62E, MCP-1, IL-12p70, IL-10, IL-8, IP-10, MIP-1α, IL-1α, IL-17A, and IL-13. In these patients, significant differences were also observed at time point B compared to point A in: MCP-1, MIP-1β, TNF-α, IL12-p70, IL-10, IL-8, IL-4, IP-10, IL-1β, IFN-γ, and IL-17A levels. Comparing cytokine levels at time point C versus point B, statistically significant differences were found in: CD26P, IL-4, and IL-1β (Supplementary Table S1 and Figure 2).

Interestingly, using multiple comparison tests, the most pronounced differences in Group 1 were observed in IL-8, IL-12p70, and MCP-1 levels at time point C versus point A. Only cytokine IL-12p70 levels significantly decreased in this group at time point B compared to time point A (Table 2).

In Group 2, the most significant differences were found in IL-12p70, IL-8, MCP-1, IL-1α, and IP-10 levels at time point C versus point A. Significant differences were also observed in IL-8, IL-12p70, and MCP-1 levels at time point B versus point A (Table 2).

Given the high variability in cytokine expressions among the included patients, an evaluation of intra-individual changes was further conducted using multiple comparison tests for all patients in both study groups to examine the levels of every cytokine at time points B and C. No significant inter-group differences were found at these time points.

Interestingly, when analysing the magnitude of change in cytokine levels at time point B versus point A (B-A) in Group 1 and at time point C versus point B (C-B) in Group 2, that is, examining only cytokine level variation that occur during the combined UT + AP treatment, statistically significant differences were found in IL-1β, IL-4, and IL-12p70 levels. Only pro-inflammatory IL-12p70 levels were down-regulated in both group of patients, although the decrease was greater in patients from Group 1 (Table 3).

Similarly, comparing the magnitude of cytokine changes at time point C versus point B (C-B) in Group 1 and at time point B versus point A (B-A) in Group 2, namely, when exploring cytokine level variation during UT alone, statistically significant differences were found in TNF-α, IL-4, IL-12p70, IL-1β, and MCP-1 levels (Figure S1 and Table 3). Of note, the changes in cytokine levels were opposite in the two groups of patients, except for MCP-1, which increased in both groups.

Finally, a comparison was made of the variations in cytokine levels between time points C and A in Group 1 and between time points B and A in Group 2 to determine if adding 6 weeks of AP prior to the UT affected the systemic inflammation state of the patients. However, no statistically significant differences were observed between both groups.

Similarly, the variations in cytokine levels between time points B and A in Group 1 were compared against the variations between points C and A in Group 2, to check if adding 6 weeks of AP to the UT resulted in differences in the variation of cytokine levels. No statistically significant differences were found in this case either.

### 2.3. Clinical Outcomes Assessment

An evaluation was conducted of the following variables to measure the functionality of patients: walking endurance and walking velocity using the 6-min walking test (6MWT) and 10-m walk test (10MWT); gait using the walking index for SCI II (WISCI II); balance using the Berg balance scale (BBS); and risk of falling with the timed up and go (TUG) test. These outcomes were recorded at the three study time points (see Figure 1, study design). The results of these analyses are shown in Table 4. Most patients in the study exhibited functional improvement upon treatment program completion, regardless of the allocation group. However, some differences can be observed in the results at the different assessment points.

**Figure 3:**
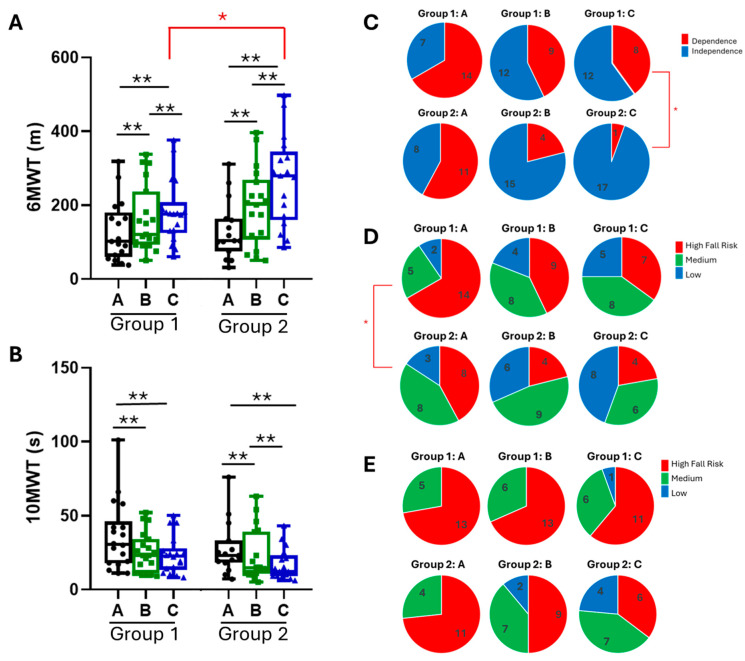
Box plots illustrating the differences in the results of 6MWT (**A**) and 10MWT (**B**) in both intervention groups. (**C**–**E**) Pie chart depicting the number of patients in each group classified according to their falling risk at the three assessments: C for WISCI II results, D for BBS results, and E for TUG test. In each figure, point A indicates the baseline, B at 6 weeks of treatment, and C at 12 weeks of treatment. * denotes *p* < 0.05; ** *p* < 0.01.

In terms of walking endurance (6MWT), the patients in Group 2 managed to travel a greater distance at time points B and C than those in Group 1. While the intergroup differences at time point B were not statistically significant, they reached statistical significance at the end of the study (point C) (*p* = 0.032, Figure 3A).

When comparing the results of the 10MWT, no statistically significant differences were observed between groups. It is worth noting that the Group 1 participants obtained their greatest improvement at point B persisting to the final assessment (point C), with no significant changes between the measurements at points B and C. However, the improvement in the 10MWT score was progressive throughout the treatment in Group 2 (Table 4 and Figure 3B).

The results of the WISCI II revealed a greater proportion of patients in Group 2 who exhibited improvements in gait at the end of the treatment (94%, 17/18) compared to Group 1 (60%, 12/20; *p* = 0.045) (Table 4 and Figure 3C).

In terms of balance, as measured with the BBS scores, no statistically significant differences were observed between both groups at points B (*p* = 0.319) or C (*p* = 0.637). Although patients in Group 1 scored worse at baseline than patients in Group 2 (*p* < 0.001), this difference was no longer evident at the subsequent assessments at time points B (*p* = 0.319) and C (*p* = 0.638) (Table 4 and Figure 3D). No statistically significant differences were found between both treatment groups in the TUG test at any of the time points of the study, although all participants exhibited improvements upon completion (Table 4 and Figure 3E).

### 2.4. Correlations between Blood Inflammatory Markers and Functional Assessment of Patients

A bivariate correlation analysis was performed to compare levels and changes in outcome variables (6MWT, 10MWT, WISCI II, BBS, and TUG tests) and blood cytokines in order to identify those achieving statistical significance or with biological plausibility based on extensive literature.

At the end of the study (time point C), inverse correlations were observed in Group 1 between: WISCI II classification and both IL-4 and TNF-α levels; 6MWT scores and IL-4 cytokine level; BBS scores and IL-8 levels; changes in the TUG test and IL-1β level; and changes on the BBS and IL-1β levels. In Group 2, an inverse correlation was observed between changes in the 10MWT (time point C—time point A) and levels of MCP-1 and IL-8.

The inverse correlation between TNF-α and WISCI II levels could already be seen at study time point B in Group 1. In Group 2 participants, a correlation was found between IL-8 levels and TUG test scores at time point B.

Table 5 shows the correlations identified, with their significance level, between the functionality variables and cytokine levels in patients from both treatment groups.

## 3. Discussion

Although extensive research has been conducted on the complex systemic inflammation following a SCI, important discrepancies remain regarding the potential influence of different physiotherapy treatments on restoring the inflammatory process balance. Nevertheless, the importance of exercise as an anti-inflammatory therapy is widely recognised [21,22,23]. People who experience a SCI are characterised by a constant, chronic, low-grade inflammatory state negatively impacting functional recovery and increasing post-injury complications. Consequently, the assessment of blood biomarkers is of major clinical importance for patient prognosis after SCI. Moreover, assessing inflammatory molecules could reveal novel potential therapeutic targets modulating the degenerative SCI processes and relevant consequences for the individual’s function. Additionally, different studies indicate that adequate and timely regulation of inflammatory reactions can take place after SCI, which would be crucial for designing therapeutic interventions that could potentially impact circulating cytokines, growth factors, or neurotrophins [13,24].

This study proposed a novel design in which AP was combined with the standard physiotherapy treatment for people with SCI. All the included patients performed the UT for 12 weeks, which was combined with AP for six of those weeks. The aim was to elucidate whether the combination of the two therapies (UT + AP), along with their specific application schedules, can affect plasma levels of inflammatory markers and consequently influence the functional recovery of these patients. This design entails a significant limitation, as the study did not include a control group of patients not receiving the combined treatment at any point. However, withholding treatment for such a control group would be unethical, especially when prescribed by their physician and considering the physical and emotional barriers of initiating therapeutic exercise that people with SCI frequently experience.

The present results corroborated the complexity of the inflammatory and reparative response occurring with SCI, as evidenced by the relationships observed between SCI and the evaluated cytokine blood levels. However, while therapeutic exercise triggered important changes in cytokine levels in patients from both treatment groups, the regulation of some of the studied cytokines must be highlighted.

The IL-12 family cytokines are key in the regulation of immune responses and inflammatory disorders. IL-12 is composed of the p35 and p40 subunits, which together form the bioactive IL-12p70. This cytokine is secreted by activated monocytes, macrophages, and dendritic cells, resulting in the activation of natural killer cells and T cells and the subsequent increased secretion of IFN-γ, IL-8, and IL-10 [25]. In the present study, a clear decrease in IL-12p70 levels at point C was observed in both treatment groups, which was already evident at point B. Of note, this effect was more pronounced in Group 1. This decrease in IL-12p70 levels may also explain the reduction in IL-8 and IL-10 levels observed in both treatment groups. Therefore, the present findings suggest blood level decrease of these pro-inflammatory cytokines could be mainly attributed to AP exercises.

Monocyte chemoattractant protein-1 (MCP-1/CCL2) is described as a chemoattractant for monocytes, natural killer cells, basophils, macrophages, neutrophils, and T and B cells involved in pathophysiological processes mediating the inflammatory immune response. The pro-inflammatory role of MCP-1 in different contexts is widely accepted [13,26]. A bioinformatics analysis by Fang et al. revealed that MCP-1 is closely related to the inflammatory response and notably upregulated after SCI [27]. However, in the present study, an increase in MCP-1 blood levels was observed at time points B and C in both treatment groups. In a different line of research using MCP-1 knockout mice, Cranford et al. reported MCP-1 deficiency exacerbated metabolic dysfunctions in their murine model, suggesting that MCP-1 may be a necessary component of the inflammatory response required for adipose tissue protection and health [28]. Therefore, although most studies recognise the pro-inflammatory effect of this cytokine after SCI, its role can vary depending on different physiological contexts.

The pro-inflammatory interferon gamma-induced protein 10 (IP-10/CXCL10) is secreted by monocytes, macrophages, and endothelial cells in response to IFN-γ. The findings of this study showed an increase in plasma IP-10 levels at time point C that was already evident at time point B in both treatment groups. However, an increase in IFN- γ levels at point B was only detected in Group 2. A systematic review by Leister et al. concluded that the serum/cerebrospinal fluid concentration of several biomarkers, including MCP-1 and IP-10, after SCI are highly time-dependent and related to injury severity [29]. Additionally, using a rat model, Hong et al. reported differences in IP-10 plasma levels depending on the spinal region of the injury [30]. Other data supports that during the acute phase after SCI and for comparable injury severity, subgroups of patients exhibited distinct circulating macrophage precursors-monocytes dominance (pro-inflammatory M1 or anti-inflammatory M2) and the phenotype was correlated with their respective specific cytokine profiles. Hence, individuals with dominant M1 showed higher circulating levels of IL12-p70 and IP-10 and lower levels of IL-10, IL-15, and IL-7, whereas those with dominant M2 exhibited the opposite cytokine profiles [31]. However, the M1 or M2 phenotypes of the participants in our study remain unknown.

Damaged microglia after SCI are ascribed to IL-1α production. In mice models, the pharmacologic abolition of the signalling pathway of this cytokine enhanced locomotor recovery after SCI [32]. In fact, this cytokine is included within the so-called alarmins or danger-associated molecular patterns, which are related to neuroinflammatory responses and secondary damage in SCI [33]. The present results revealed a statistically significant decrease in IL-1α levels at the end of both physiotherapy programs, which is consistent with a potential reduction in the inflammatory state of the included patients.

Interestingly, the findings showed an increase in the levels of pro-inflammatory cytokines MCP-1 and IP-10. One potential explanation could be an increase in the number of immune cells in response to exercise, but we were unable to verify this effect in our study. Similarly, at the end of both treatments, a decrease in the levels of anti-inflammatory cytokines IL-10 and IL-13 was observed, although without reaching statistical significance. A possible explanation could be that following the initial pro-inflammatory systemic state, cytokines return to normal levels as part of the blood homeostatic system functioning. The analysis conducted did not reveal differences in IL-6 levels at the different assessments of the study, even if this cytokine is one of the most characteristic ones of the inflammatory response and stimulates the exercise-driven anti-inflammatory cascade. However, former research reported a certain exercise intensity needs to be reached to initiate increasing anti-inflammatory cytokines, and the participants in this study may not have attained this exercise level threshold [23].

One of the most important uses of biomarkers in the clinical setting could be to predict functional recovery and to identify those patients with high motor improvement likelihood. Therefore, an analysis was performed of the relationship between cytokine levels and five validated tests for the functional evaluation of SCI patients. As expected, most patients had improved motor abilities with therapy intervention. However, participants in Group 2 obtained greater improvements in the 6MWT, WISCI II, and TUG tests, while participants in Group 1 exhibited greater improvement in the 10MWT immediately after finishing the combined therapy of AP + UT (time point B), which was maintained until the post-intervention assessment (time point C). The inter-group comparison of balance (BBS scores) should be taken with caution, given the significant differences between both groups at the baseline (time point A). However, the results at the treatment end showed balance improvements of similar magnitude in both groups, which may be indicative of the impact early aquatic exercise initiation can have for functional recovery. In view of these results, we cannot conclude which of the two intervention schedules is more beneficial for SCI patients. However, the incorporation of AP into the UT appears to yield greater functional gains in patients with SCI, and this improvement seems to weaken when AP is abandoned. Reported improvement in physical function and activities of daily living related to AP exist in previous research [7,8]. Nevertheless, more studies with larger sample sizes are necessary to validate these findings.

In Group 1, inverse correlations were found at the end of treatment between: IL-4 levels and the 6MWT and WISCI II outcomes; TNF-α level and WISCI II classification; and IL-8 level and BBS scores. Additionally, in Group 2, an inverse correlation was found at the end of the treatment between levels of MCP-1 and IL-8 and changes in the 10MWT. To our knowledge, this is the first analysis correlating cytokine levels and functional improvement in SCI patients following different treatment programs. Furthermore, broader studies on cytokine blood levels along with proteomic, metabolomics, and genetic studies would enable a more comprehensive understanding of the acute pathophysiology of SCI and the identification of additional biomarkers that may be helpful in predicting outcomes.

This study includes several limitations. First, the number of participants included is relatively small. However, recruiting larger samples of individuals with SCI may prove difficult considering that SCI is a rare, highly heterogeneous disorder. Patients were recruited in a single, relatively small public hospital specialised in the comprehensive treatment of SCI (National Hospital for Paraplegics, 222 beds), which hindered the inclusion of a large number of patients. Also, the UT prescribed differed among participants, which could create potential cytokine level variability. Finally, this study did not consider the pharmacological treatment specific to each patient.

In summary, this study identified a complex signature of five cytokines (IL-12p70, IL-8, MCP-1, IL-1α, and IP10) that were associated with the time course of two different physiotherapy treatments in SCI patients. Two additional cytokines, IL-4 and TNF-α, were also related to the functional recovery of patients. These findings can serve to establish guidelines and intervention protocols to optimise the rehabilitation of SCI patients, as inflammation is a relevant process in the pathophysiology of this condition. Therefore, regulating systemic inflammation would decrease tissue and functional damage and favour the recovery of functions lost due to the injury. However, depending on the therapeutic strategy after SCI, the inflammatory response and the functional effects can be affected differently.

The present work highlights the importance of developing new physiotherapy strategies that regulate the inflammatory response by focusing on target cytokines as a key monitor of functional recovery after SCI. However, further research is warranted to develop an effective and accurate regulation of inflammation, as well as intervention schedules that include AP, to potentially influence the recovery of individuals with SCI.

## 4. Materials and Methods

### 4.1. Study Design and Patients

This single-centre prospective study enrolled 50 inpatients with SCI of less than six months of evolution who were being treated at the National Hospital for Paraplegics (Toledo, Spain) between November 2021 and December 2023. Subjects who agreed to participate were randomized into two groups using a table generated with Epidat© statistical software version 3.1. Among the selected participants, three patients voluntarily withdrew from the study, two patients were discharged from the hospital before completing the treatment, one patient was withdrawn by their physician due to a recurrent urinary infection, and a sufficient blood sample could not be obtained at any of the time points in four other patients.

Eligible participants with SCI were 18 to 70 years old, classified as C or D according to the AIS, had an injury of less than six months of evolution, had achieved assisted standing at least one week before enrolment, and were receiving physiotherapy treatment during hospitalisation. Patients with other diseases involving the immune system, severe infections, or open skin lesions, and/or cardiovascular, pulmonary, or metabolic diseases were excluded.

Demographic information (age and sex), anthropometric measures (height and weight), socioeconomic data (employment status, educational level), and clinical information (injury type, AIS classification) were obtained from medical records or through patient interviews. Blood samples of adequate quality and volume could only be obtained at all the scheduled assessments from 40 of the patients initially included. The analyses were conducted using the data from this final sample.

The study has been registered in ClinicalTrial.gov with ID: NCT03962218.

### 4.2. Exercise Protocols

The participants performed the UT on the ground throughout the complete study period, consisting of standard physical treatment including joint mobilisation below the level of the injury, strengthening of the supralesional musculature and the remaining motor functions, muscle stretching and postural relaxation techniques to treat spasticity, trunk stabilisation, and practising self-care strategies.

The AP program included 18 sessions, 3 days per week, with a duration of 40 min per session. This therapy consisted of water-based exercises in a 1.20 m deep pool. Exercise intensity was monitored using the rating of perceived effort (RPE) scale. The intervention was water-based exercises incorporating water specific therapy (RPE 10–11), clinical Ai-chi (RPE 8–9), and aerobic exercise at 60–70% of max. heart rate (RPE 13–15) [34,35,36].

Group 1 performed AP combined with UT for the first six weeks of the intervention, while Group 2 performed the combined AP and UT program throughout the last six weeks (Figure 1).

### 4.3. Multiplex Immunoassay

Blood samples were collected in EDTA tubes before starting therapy intervention (time point A), after six weeks (point B), and post-intervention (point C). The samples were immediately centrifuged for 5 min, at 2,000 rpm at room temperature, and the plasma was stored at −80 °C.

A Luminex magnetic bead commercial assay panel (Human ProcartaPlex™ Inflammation Panel; Thermo Fisher Scientific (Vienna, Austria)) was employed to measure circulating levels of a wide array of cytokine and inflammation markers, specifically: cytokines (GM-CSF, IFNα, IFNγ, IL-1α, IL-1β, IL-4, IL-6, IL-8, IL-10; IL-12p70, IL-13, IL-17A (CTLA-8), and TNFα), chemokines (IP10 (CXCL10), MCP-1 (CCL2), MIP-1α (CCL3), and MIP-1β (CCL4)), and cell adhesion molecules (ICAM-1, CD62E (E-selectin), and CD62P (P-selectin)). All samples were analysed in duplicate. The multiplex panel was operated following the manufacturer procedures manual and analysed using a Luminex 200™ reader (Merck KGaA, Darmstadt, Germany). The quantitative determination of the relevant analytes was achieved by comparing the raw data obtained from the plasma samples with a standard curve.

### 4.4. Functional Assessment of Patients

The functional evaluation of the patients throughout the study was performed using five validated tests. The 6MWT measures the maximum distance walked by a person on a flat surface in 6 min. The 10MWT is a performance measure of the time taken to walk a distance of 10 metres without assistance [37]. The WISCI II consists of an ordinal scale including 19 items to record the type and degree of assistance SCI patients require for walking [38]. The BBS contains 14 items to evaluate balance [39]. Finally, the timed up and go (TUG) is a test of basic functional mobility that examines the time a patient takes to get up from a chair, walk 3 metres at their usual pace, turn around, return to the chair, and sit down [40]; two attempts are made, and the best time is selected for analysis.

### 4.5. Statistical Methods

All analyses were performed using SPSS software v28.0 (SPSS Inc., Armonk, NY, USA) and graphics were generated with GraphPad Prism v8.0.1 (San Diego, CA, USA). All data were quantified at time points A, B, and C, as established in the study schedule. Variations in cytokine levels and functional parameters were estimated through simple change levels (Δ) between time points AC, AB, and BC. The distribution of data was examined using the Shapiro–Wilk test. The chi-squared or Fisher’s exact tests were used for analysing categorical data. Inter-group comparisons were assessed using the independent or paired *t*-tests together with the Levene’s test to assess the equality of variance. Whenever the data did not fit a normal distribution, the Mann–Whitney U and Wilcoxon tests were used for inter-group comparison. The identification of differentially expressed cytokines by multiple comparison procedures was achieved using the two-stage linear step-up method of Benjamini, Krieger, and Yekutieli with Q = 1%. To this end, each cytokine level was analysed individually, without assuming a consistent SD. Multiple comparison correction was performed and the q-value is indicated in Table 3. 

The q-value is defined as the expected proportion of false positives among all tests equal to or more extreme than the one observed. The two-tailed Pearson and Spearman’s correlation coefficients were employed to explore the relationship between cytokine levels and functional outcomes for normally or non-normally distributed data, respectively. Statistical significance was set at *p* < 0.05.

## Figures and Tables

**Table 1:** Socioeconomic and clinical characteristics of the patients included in the study.

	Group 1 (N = 21)	Group 2 (N = 19)	*p*-Value
Sex (men/women)	9/12	13/6	0.096 ^1^
Age (years; median, Q1–Q3)	50.0 (39.5–60.0)	58.0 (38.0–61.0)	0.390 ^2^
Marital status (N, %)			0.019 ^3^
Single	8 (38.1)	3 (15.8)	
Married	6 (28.6)	14 (73.7)	
Divorced/Widowed	7 (33.3)	2 (10.5)	
Employment status (N, %)			0.262 ^3^
Employed	17 (80.9)	11 (57.8)	
Unemployed	1 (4.8)	4 (21.1)	
Retired	3 (14.3)	4 (21.1)	
Education level (N, %)			0.288 ^3^
Primary	6 (28.6)	9 (47.4)	
Secondary	12 (57.1)	6 (31.6)	
University	3 (14.3)	4 (21.0)	
BMI (Kg/m^2^; median, Q1–Q3)	25.76 (21.4–29.3)	25.54 (23.3–29.7)	0.872 ^2^
Injury type (N, %)			0.500 ^3^
Traumatic	10 (47.6)	10 (52.6)	
Non-traumatic	11 (52.4)	9 (47.4)	
AIS (N, %)			0.181 ^3^
C	14 (66.7)	9 (47.4)	
D	7 (33.3)	10 (52.6)	
Time from injury (months)	3.6 ± 1.3	3.1 ± 0.8	0.06 ^1^

^1^ Fisher’s exact test. ^2^ Mann–Whitney test. ^3^ Chi-squared test. N: number of subjects. BMI: body mass index. AIS: ASIA Impairment Scale: C = motor incomplete; sensory function preserved at the most caudal sacral segments and less than half of key muscle functions below the single neurological level of injury with a muscle grade ≥ 3. D = motor incomplete; sensory function preserved at the most caudal sacral segments and at least half of key muscle functions below the single neurological level of injury with a muscle grade ≥ 3.

**Table 2:** Statistically significant effects on cytokine levels by multiple comparison (two-stage linear step-up method of Benjamini, Krieger, and Yekutieli).

		A	B	C	SE of Difference	*p*-Value	q-Value
Group 1	IL-8	10.94		5.866	0.829	<0.000001	0.000006
	IL-12p70	324.2		205.1	25.42	0.000033	0.000270
	MCP-1	79.6		123.9	12.21	0.000815	0.004392
	IL-12p70	324.2	205.1		16.72	<0.000001	<0.000001
Group 2	IL-12p70	339.6		221.6	18.58	<0.000001	0.000003
	IL-8	10.57		6.681	0.695	0.000003	0.000017
	MCP-1	72.46		120.7	10.40	0.000048	0.000211
	IL-1α	2.962		1.926	0.260	0.000327	0.000936
	IP-10	31.74		51.19	4.917	0.000356	0.000936
	IL-8	10.57	6.131		0.696	<0.000001	0.000004
	IL-12p70	339.6	232.5		20.81	0.000010	0.000077
	MCP-1	72.46	107.4		8.681	0.000283	0.001527

Columns: time point A at the baseline, time point B at 6 weeks of treatment, time point C at 12 weeks of treatment. Group 1 first received 6 weeks of AP combined with UT followed by 6 weeks of UT alone. Group 2 first received 6 weeks of UT followed by 6 weeks of UT + AP joint treatment. SE: standard error.

**Table 3:** Statistically significant inter-group differences found via multiple comparison tests in cytokine changes at the end of combined treatment (UT + AP) or at the end of UT.

		*p*-Value	Mean Change Group 1	Mean Change Group 2	SE of Difference	q-Value
(B-A) Group 1 vs. (C-B) Group 2 (UT + AP)	IL-1β	0.00082	−10.71	2.513	3.63	0.0044
	IL-12p70	0.00004	−119.1	−7.277	23.75	0.00056
	IL-4	0.00047	5.576	−13.68	5.023	0.00383
(C-B) Group 1 vs. (B-A) Group 2 (UT)	IL-1β	0.00031	3.727	−7.448	2.810	0.0011
	IL-12p70	0.00123	7.474	−107.1	26.72	0.00058
	IL-4	0.00003	−13.09	10.8	5.048	0.00023
	TNF-α	0.00003	−12.87	15.89	6.071	0.00023
	MCP-1	0.00076	7.861	34.91	7.372	0.00216

SE: standard error.

**Table 4:** Assessment of patients’ functionality throughout the study.

			A	B	C
Group 1	6MWT (m; median, Q1–Q3)		101.8	120.8	175.9
			(60.1–180.3)	(92.4–236.6)	(124.4–207.7)
	10MWT (m/s; median, Q1–Q3)		0.33	0.43	0.44
			(0.24–0.56)	(0.29–0.91)	(0.36–0.76)
	WISCI-II (%)	Dependence	14/21 (66.7)	9/21 (42.9)	8/20 (40.0)
		Independence	7/21 (33.3)	12/21 (57.1)	12/20 (60.0)
	BBS (%)	High	14/21 (66.7)	9/21 (42.9)	7/20 (35.0)
		Medium	5/21 (23.8)	8/21 (38.1)	8/20 (40.0)
		Low	2/21 (9.5)	4/21 (19.0)	5/20 (25.0)
	TUG (%)	High	13/18 (72.2)	13/19 (68.4)	11/18 (61.1)
		Medium	5/18 (27.8)	6/19 (31.6)	6/18 (33.3)
		Low	0/18 (0)	0/19 (0)	1/18 (5.6)
Group 2	6MWT (m; median, Q1–Q3)		103.6	203.4	279.2
			(75.0–163.2)	(106.7–268.4)	(159.8–344.9)
	10MWT (m/s; median, Q1–Q3)		0.43	0.69	0.83
			(0.28–0.61)	(0.25–0.93)	(0.44–1.11)
	WISCI-II (%)	Dependence	11/19 (57.9)	4/19 (21.1)	1/18 (5.6)
		Independence	8/19 (42.1)	15/19 (78.9)	17/18 (94.4)
	BBS (%)	High	8/19 (42.1)	4/19 (21.0)	4/18 (22.2)
		Medium	8/19 (42.1)	9/19 (47.4)	6/18 (33.3)
		Low	3/19 (15.8)	6/19 (31.6)	8/18 (44.5)
	TUG (%)	High	11/15 (73.3)	9/18 (50.0)	6/17 (35.3)
		Medium	4/15 (26.7)	7/18 (38.9)	7/17 (41.2)
		Low	0/15 (0)	2/18 (11.1)	4/17 (23.5)

Columns: time point A at the baseline, time point B at 6 weeks of treatment, time point C at 12 weeks of treatment. The outcome data are expressed by the number of cases out of the total number of patients analysed unless otherwise specified. 6MWT: 6-min walking test. 10MWT: 10-m walk test. WISCI-II: Walking index for spinal cord injury II. A test score ≤ 9 indicates dependence and ≥10 indicates independent gait. BBS: Berg balance scale; test results ≤ 20 indicate high risk of falling, 21–40 indicate medium risk, and 41–56 low risk. TUG: timed up and go; test timings > 20 s indicate high risk of falling, 10–20 s indicate medium risk, and <10 s low risk.

**Table 5:** Correlations between changes in outcome measures and variations in cytokine blood levels and comparison between both treatment groups.

	Group 1		Group 2	
	Correlation	*p*-Value	Correlation	*p*-Value
Time point C				
IL-4/6MWT	ρ = −0.484	0.042	r =− 0.002	0.993
IL-4/WISCI	r = −0.541	0.014	r = 0.162	0.520
TNF-α/WISCI	r = −0.507	0.023	r = −0.062	0.806
IL-8/BBS	r = −0.464	0.04	ρ = 0.202	0.424
IL-8/ΔBBS ^1^	r = −0.445	0.044	ρ = 0.239	0.340
IL-1β/ΔTUG ^1^	ρ = −0.511	0.036	r = 0.103	0.715
MCP-1/Δ10MWT ^1^	ρ = −0.109	0.668	r = −0.679	0.015
IL-8/Δ10MWT ^1^	ρ = −0.147	0.561	ρ = −0.613	0.034
ΔIL-8/Δ10MWT ^1^	ρ = −0.119	0.639	r = −0.776	0.003
IL-1α/Δ6MWT ^2^	ρ = −0.496	0.036	r = 0.187	0.472
IP10/Δ10MWT ^2^	ρ = 0.627	0.005	ρ = −0.132	0.613
Time point B				
TNF-α/WISCI	ρ = −0.479	0.028	ρ = −0.023	0.925
IL-8/TUG	ρ = −0.377	0.111	ρ = −0.549	0.018
IL-1β/Δ6MWT ^3^	r = 0.160	0.539	r = −0.533	0.041
IL-4/Δ6MWT ^3^	r = −0.039	0.883	r = −0.528	0.043
IL-4/ΔWISCI ^3^	r = 0.064	0.782	ρ = −0.474	0.040
TNF-α/ΔWISCI ^3^	r = −0.115	0.62	ρ = −0.492	0.033
IL-1β/ΔTUG ^3^	r = −0.132	0.602	r = 0.660	0.007
IL-1 α/ΔTUG ^3^	r = 0.008	0.975	r = 0.543	0.036
IL-12p70/ΔTUG ^3^	ρ = −0.301	0.225	r = 0.614	0.015
IP-10/ΔTUG ^3^	ρ = 0.051	0.842	ρ = −0.551	0.033
ΔIL-12p70/Δ6MWT ^3^	ρ = −0.054	0.837	r = −0.710	0.003
ΔIL-4/Δ6MWT ^3^	r = −0.173	0.507	r = −0.561	0.029
ΔIL-1α/Δ6MWT ^3^	r = −0.030	0.909	r = −0.689	0.005
ΔIL-8/ΔWISCI ^3^	ρ = −0.488	0.025	ρ = −0.044	0.844

Only statistically significant correlations are shown; r: Pearson correlation coefficient; ρ: Spearman’s correlation coefficient. Δ: change. ^1^ Changes from the beginning to the end of the study (C-A). ^2^ Changes from time point B to the end of the study (C-B). ^3^ Changes from the beginning to time point B of the study (B-A).

## Data Availability

The raw data supporting the conclusions of this article will be made available from the corresponding author upon reasonable request.

## Supplementary Materials

The following supporting information can be downloaded at: https://www.mdpi.com/article/10.3390/ijms25147961/s1.
