# Supplementary material for: Systemic Inflammatory Changes in Spinal Cord Injured Patients after Adding Aquatic Therapy to Standard Physiotherapy Treatment
Source: Int J Mol Sci. 2024 Jul 21;25(14):7961. doi: 10.3390/ijms25147961 (PMC11277190; doi:10.3390/ijms25147961)
Supplement: Supplementary file 1 [file ijms-25-07961-s001.zip › ijms-3079450-supplementary.pdf]

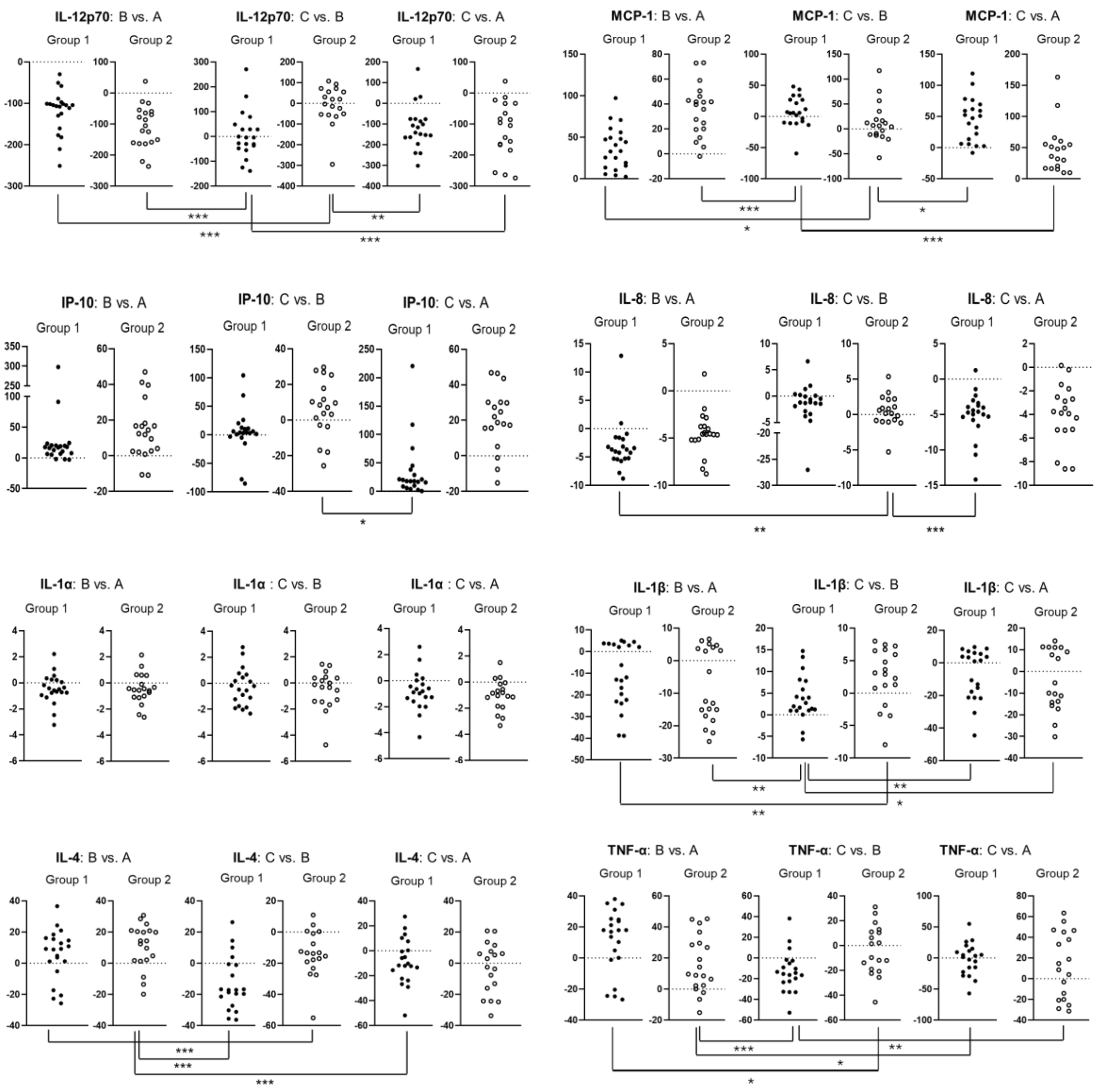

Figure S1. More representative changes in cytokines that appear modulated at different time points, compared one to one, throughout the physiotherapy treatment of patients in groups 1 and 2. Cytokines are measured in pg/ml. Spot on the graphs represent variations intra-individuals (above the dashed line of 0, increases; and below the dashed line, decreases). *: $P< 0.05$; **: $P<0.01$; ***: $P<0.001$.

Supplementary Table S1: Changes in cytokines levels between time points, in Group 1 and Group 2 of patients.

	Group 1				Group 2			
	**C vs.A**	**B vs. A**	**C vs.B**	***P*-value**	**C vs.A**	**B vs. A**	**C vs.B**	***P*-value**
CD26P(x10³)	386.2±523.7			0.0038	683.2±799.3			0.0021
CD62E(x10³)	5.3±5.9			0.0008	4.0±3.7			0.0003
MCP-1	44.7±35.1			<0.0001	47.5±39.2			<0.0001
IL-12p70	-116.1±104.4			<0.0001	-117.1±90.6			<0.0001
IL-10	-4.1±5.2			0.0023	-3.2±5.5			0.0267
IL-8	-5.1±3.3			<0.0001	-4.0±2.6			<0.0001
IP-10	35.5±51.5			0.0061	20.5±17.3			0.0001
MIP-1α					-2.3±4.4			0.0402
IL-1α	-0.81±1.5			0.0234	-0.99±1.2			0.0027
IL-17A					7.6±12.5			0.0197
IL-13	-3.5±4.9			0.0057	-2.4±4.1			0.0224
CD26P (x10³)		143.7±156.4		0.0004				
CD62E (x10³)		3.6±4.8		0.0026				
MCP-1		37.0±25.5		<0.0001		34.9±21.1		<0.0001
MIP-1β		14.0±22.2		0.0091		12.2±21.9		0.0258
TNF-α		11.3±20.4		0.0202		15.9±18.2		0.0013
GM-CSF		-25.5±29.9		0.0009				
IL12-p70		-119.1±52.3		<0.0001		-107.1±69.2		< 0.0001
IL-10						-3.4±4.1		0.0023
IL-8		-3.1±4.3		0.0036		-4.4±2.3		<0.0001
IL-4						10.8±14.2		0.0038
IP-10		30.0±64.3		0.0453		14.1±16.4		0.0014
IL-1β		-10.7±14.8		0.0035		-7.4±11.4		0.0106
IFN-γ								0.0126
IL-17A		5.6±8.1		0.0053		5.3±9.2		0.0226
IL-13		-3.0±2.7		<0.0001				
CD26P (x10³)			239.5±462.2	0.0318			627.2±798.8	0.004
IL-1 β			3.7±5.2	0.0045			2.5±4.4	0.0274
IL-4			-13.1±17.1	0.0029			-13.7±14.9	0.0012
TNF-α			-12.9±19.6	0.0085				

Data are shown as Means of differences ± SD of differences (paired t-test). Values are pg/mL. Time point A at the baseline, time point B at 6 weeks of treatment, time point C at 12 weeks of treatment. Group 1 first received 6 weeks of AP combined with UT followed by 6 weeks of UT alone. Group 2 first received 6 weeks of UT followed by 6 weeks of UT+AP joint treatment.
